# Dynamics of ethylene glycol-based graphene and molybdenum disulfide hybrid nanofluid over a stretchable surface with slip conditions

**DOI:** 10.1038/s41598-022-05703-z

**Published:** 2022-02-02

**Authors:** Syed M. Hussain

**Affiliations:** Department of Mathematics, Faculty of Science, Islamic University of Madinah, Medina, 42351 Saudi Arabia

**Keywords:** Engineering, Mathematics and computing, Nanoscience and technology

## Abstract

In this research study, numerical and statistical explorations are accomplished to capture the flow features of the dynamics of ethylene glycol-based hybrid nanofluid flow over an exponentially stretchable sheet with velocity and thermal slip conditions. Physical insight of viscous dissipation, heat absorption and thermal radiation on the flow-field is scrutinized by dissolving the nanoparticles of molybdenum disulfide (MoS_2_) and graphene into ethylene glycol. The governing mathematical model is transformed into the system of similarity equations by utilizing the apt similarity variables. The numerical solution of resulting similarity equations with associated conditions are obtained employing three-stages Lobatto-IIIa-bvp4c-solver based on a finite difference scheme in MATLAB. The effects of emerging flow parameters on the flow-field are enumerated through various graphical and tabulated results. Additionally, to comprehend the connection between heat transport rate and emerging flow parameters, a quadratic regression approximation analysis on the numerical entities of local Nusselt numbers and skin friction coefficients is accomplished. The findings disclose that the suction and thermal radiation have an adverse influence on the skin friction coefficients and heat transport rate. Further, a slight augmentation in the thermal slip factor causes a considerable variation in the heat transport rate in comparison to the radiation effect.

## Introduction

Recently, an innovative class of nanotechnology is developed with better chemical and thermal features by hybrid nanofluids. A hybrid nanofluid is prepared by the dispersal of two or more nano-sized metal or metal oxides into a conventional (base) fluid. The thermophysical features and significance of nanoparticles of various metals (Cu, Au, Ag, etc.), metal oxides (Al_2_O_3_, ZnO, TiO_2_, SiO_2_, MoS_2_, etc.), metal nitrides (Boron nitride BN, AIN), metal carbides (SiC), carbon materials (CNTs, graphene, diamonds, etc.) and hybrid nanomaterials are available in the literature^1^. Each nanoparticle has inimitable thermal features and is utilized as per the need of the thermal systems. In this research exploration, the nanoparticles of graphene and molybdenum disulfide (MoS_2_) are disseminated into the ethylene glycol to prepare the hybrid nanofluid. The nanoparticles of graphene and molybdenum disulfide (MoS_2_) have tremendous thermal performance and are applicable in various thermal systems^2^. The graphene (allotrope of carbon) nanoparticles own the unique material, chemical, electrical and physical characteristics because it has a single layer of atoms, biocompatibility certainties, expanded surface area, cell growth capability, fast mobility of electrons, stability, and high thermal conductivity. On the other hand, molybdenum disulfide (MoS_2_) consists of a layered structure and has distinctive properties such as chemically inertness, photo corrosion resistance and anisotropy, etc. Experimental findings revealed that hybrid nanofluids have better efficiency as compared to nanomaterials. Novel characteristics of hybrid nanofluids are significant in thermal storage, solar heating, transformer cooling, biomedical industry, heat pumps, refrigeration, welding, aircraft, spacecraft, lubrication, generator and electronic cooling, etc. Suresh et al.^3^ quantified the mechanism of thermal transport on the dynamics of water conveying Cu-Al_2_O_3_ hybrid nanofluid. They analysed the significant improvement in convective heat transfer owing to synthesized hybrid particles’ addition as compared to water. Devi and Devi^4^ implemented the Runge-Kutta-Fehlberg algorithm to analyse the heat transport rate of three-dimensional water-driven Cu-Al_2_O_3_ nanoparticles hybrid nanofluid flow through a stretchable surface under the inspiration of suction and Lorentz force. The numerical findings disclosed that the heat transport rate of water conveying Cu-Al_2_O_3_ nanoparticles hybrid nanofluid is better than Cu-water nanofluid. Further, in the extended work, Devi and Devi^5^ showed that the heat transport rate of water-driven Al_2_O_3_-Cu hybrid nanofluid can be improved to 17.3% as compared to base liquid water whereas 11.2% than the Cu-water nanofluid. Recently, Khashi’ie et al.^6^ enumerated the significance of the magnetic field and suction strength on the time-dependent squeezing flow of water-based Al_2_O_3_-Cu hybrid nanofluid through a parallel channel. They observed that the heat transport rate of the hybrid nanofluid at the lower plate can be reduced due to the inclusion of injection, squeezing and magnetic parameters while it can be improved at the upper plate of the channel nearly by 30.35% owing to the augmentation of suction and magnetic field strength.

Because of the applications in industry, the novel features of thermal radiation cannot be overlooked exclusively in the designing of steadfast equipment, furnaces, electrical power generation, nuclear plants, gas turbines, satellites, missiles, and also in designing of various cutting-edge energy conversion systems. These days, owing to the diminution of traditional energy sources, researchers are giving much attention to renewable energy resources. Solar energy is the fundamental basis of renewable energy and the thermal effect performs as an important fragment to change solar energy to the apt form for different industrial applications. Keeping the significance of radiation effect into account, the influence of nonlinear radiation on the Cu-Al_2_O_3_ nanoparticles driven flow of micropolar dusty hybrid nanofluid via a stretchable surface was scrutinized by Ghadikolaei et al.^7^. The nonlinear radiation impact on the thermal conductive flow of hybrid nanofluid (water/Cu-Al_2_O_3_) through a three-dimensional stretchable surface in a rotating medium using the least square approach was surveyed by Usman et al.^8^. Further, Sheikoleslami et al.^9^ inspected the non-Darcy model of hybrid nanofluid considering Hartmann effects and a radiation term. They analysed that the temperature gradient gets highly affected owing to augmenting values of buoyancy parameter. Shoaib et al.^10^ studied the three-dimensional rotating flow of magneto-hybrid nanofluid past an extendable surface with thermal radiation. Some illustrious research articles highlighting the novel features of thermal radiation can be seen in references^11–18^. In addition, the inclusion of heat generation/absorption is important in controlling the heat transport rate in several industrial processes such as in glass fibre production, hot rolling, endothermic reactions, paper production, heat conversation due to nuclear fuel wastage, etc. Following this, Hayat and Nadeem^19^ elucidated the implication of heat absorption/generation on the heat transport features of three-dimensional chemically reactive and radiative hybrid nanofluid flow through a stretchable surface. They concluded from their numerical exploration that the inspirations heat generation, radiation and chemical reaction are significant to achieve the higher heat transport rate of hybrid nanofluid as compare to simple nanofluid. This numerical exploration was further extended by Hayat et al.^20^ to capture the effects of heat generation/absorption, considering the hybrid nanofluid in a rotating medium. Recently, Li et al.^21^ assessed the mathematical model to illustrate the effect of heat generation/absorption on the entropy optimised convective flow in a rotating cone. They concluded that the upsurge in viscosity parameter and buoyancy ratio variable result in a significant rise in the tangential velocity.

One point to note here is that, in all the research articles reported above, the majority of authors have overlooked viscous dissipation term because of the feeble result on the flow field but its bearing in polymer manufacturing, lubrication, instrumentations, food processing, etc., is substantial because it augments the temperature distribution features and subsequently upsurges the heat transport rate. Few novel research articles describing the implication of viscous dissipation on various flow problems persuaded by a stretchable surface/thin stirring needle are cited in references^22–25^. In addition, Joule dissipation demonstrates the features of volumetric heat source in magneto-fluid flows and combined Joule and viscous dissipations inspirations are noteworthy in various heat-treated materials. Owing to this reason, Seth et al.^26^, Daniel et al.^27^ and Seth and Singh^28^ taken both the terms of Joule and viscous dissipations together in their mathematical models. Flow features of propylene glycol conveying, entropy optimized, magneto and dissipative Darcy-Forchheime nanofluid via a stretchable surface was illustrated by Abbas et al.^29^. Further, Wang et al.^30^ explored the irreversibility features of entropy optimized magneto-nanofluid flow via variable thick surface under inspirations of viscous dissipation and Joule heating. Numerical exploration was done by Ibrahim and Khan^31^ to analyse the influence of viscous dissipation on SWCNT and MWCNT conveying mixed convection nanofluid flow. Slip conditions are being extensively inspected by numerous researchers owing to their importance in the heat transport process which occurs due to the dissimilar fluid velocity and the velocity of fluid near the boundary. In industry, slip condition is widely used in micro heat exchangers, microelectronics cooling devices, polishing, artificial heart valves, internal cavities and drug delivery system^32–34^. Lately, Hussain et al.^35^ inspected the flow of graphene and ethylene glycol-based Maxwell nanofluid via a stretchable surface under the inspirations of thermal radiation, slip conditions, viscous and Joule dissipations. This problem was further extended by Sharma et al.^36^ considering heat absorption into account. The numerical findings divulged that viscous dissipation, radiation and slip parameters are important in controlling the temperature of the flow field. Also, heat transport rate is more sensitive to radiation parameters in contrast with viscous dissipation. Very recently, Wahid et al.^37^ investigated the dynamics of hybrid nanofluid flow in the presence of velocity slip and heat generation via an exponentially stretchable surface. They analysed that the shear stress and wall temperature gradient can be augmented by enhancing the volume fraction of copper nanoparticles. Recent additions considering nanofluids with heat and mass transfer in various physical situations are given by Refs.^38–53^.

Encouraged from the available literature, in this research exploration, we envisioned examining the dynamics of ethylene glycol-based hydromagnetic hybrid nanofluid containing graphene and MoS_2_ nanoparticles over an exponentially stretchable sheet with partial slip and thermal jump conditions. The significance of viscous dissipation, heat absorption and thermal radiation on the flow field is scrutinized under both velocity slip and without slip conditions. The governing mathematical equations of the problem are solved numerically with the help of three stages Lobatto IIIa-bvp4c solver based on a finite difference scheme in MATLAB. Moreover, to comprehend the connection between heat transfer rate and emerging flow parameters, a statistical method is accomplished for the quadratic regression approximation on the numerical entities of local Nusselt numbers and skin friction coefficients. Finally, the computed numerical results are compared with the published results under limited conditions to validate the numerical solution.

## Mathematical foundation of the problem

**Figure 1:**
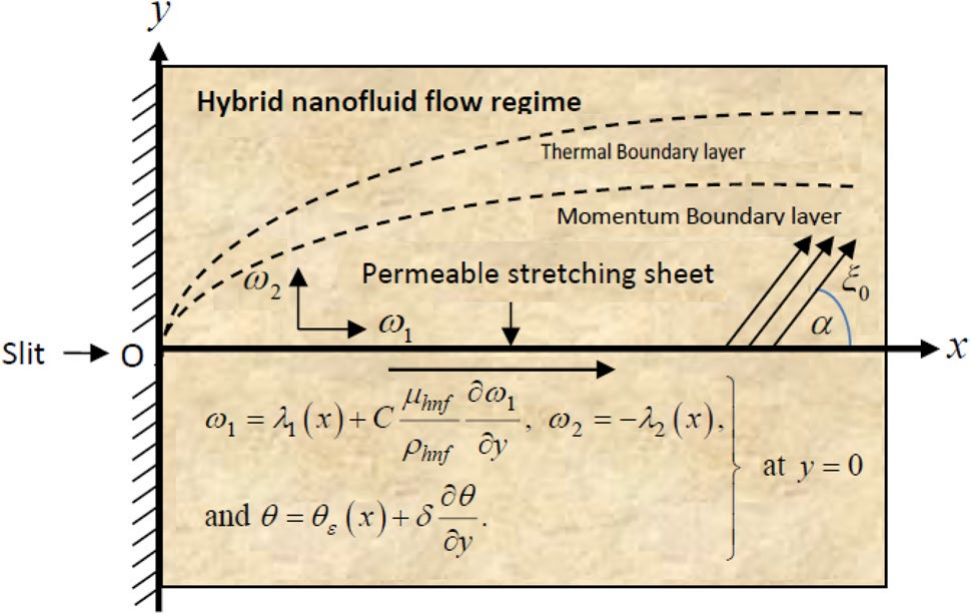
Physical representation of the problem.

In this numerical exploration, the dynamics of two-dimensional, steady-state, incompressible and electrically conducted flow of ethylene glycol-based hybrid nanofluid over an exponentially stretchable sheet under the stimulus of partial slip and thermal jump conditions are examined. The base fluid i.e., ethylene glycol is hybridized by immersing very fine nanoparticles of molybdenum disulfide (MoS_2_) and graphene into the fluid. For the development of the mathematical model, the Cartesian coordinate system is considered where the *x*-axis is allied with the surface of the stretchable sheet, the *y*-axis is taken in a perpendicular direction and the flow-field is constrained to $y > 0$. A physical diagram of the problem is exhibited in Fig. 1. The following assumptions are made to obtain the governing equations for the dynamics of hybrid nanofluid flow:

- An unvarying oblique magnetic field $\xi_{0}$ is imposed in a direction that makes an angle $\alpha$ with the stretchable sheet, which is adequately weak to ignore the induced magnetic field Cramer and Pai^54^.
- The hybrid nanofluid is imbued over the stretchable surface owing to the ambient velocity $\lambda_{1} \left( x \right)$.
- The base fluid ethylene glycol and the nanoparticles of MoS_2_ and graphene are in a thermal equilibrium state and no-slip befalls between them.
- The polarization impact is overlooked owing to the non-appearance of the externally exerted electric field.
- Inspirations of heat absorption, viscous dissipation and optically thick radiation are unified to improve the heat transport rate.
- The temperature of hybridized fluid at the stretchable sheet is $\theta_{\varepsilon }$ while those of hybrid nanofluid is $\theta_{\infty }$.

Based on the aforesaid assumptions, the constitutive flow equations of momentum and energy for the ethylene glycol-based hybrid nanofluid are obtained as^55^:

The continuity equation:

1 $$\frac{{\partial \omega_{\,1} }}{\partial x} + \frac{{\partial \omega_{\,2} }}{\partial y} = 0.$$

The momentum equation:

2 $$\omega_{\,1} \frac{{\partial \omega_{\,1} }}{\partial x} + \omega_{\,2} \frac{{\partial \omega_{\,1} }}{\partial y} = \frac{{\hat{\mu }_{hnf} }}{{\hat{\rho }_{hnf} }}\frac{{\partial^{2} \omega_{\,1} }}{{\partial y^{2} }} - \frac{{\hat{\sigma }_{hnf} }}{{\hat{\rho }_{hnf} }}\xi_{0}^{2} \,\sin^{2} \alpha \,\omega_{\,1} .$$

The energy equation:

3 $$\begin{aligned} \omega_{\,1} \frac{\partial \theta }{{\partial x}} + \omega_{\,2} \frac{\partial \theta }{{\partial y}} & = \frac{{\hat{K}_{hnf} }}{{\left( {\hat{\rho }C_{p} } \right)_{hnf} }}\frac{{\partial^{2} \theta }}{{\partial y^{2} }} - \frac{1}{{\left( {\hat{\rho }C_{p} } \right)_{hnf} }}\frac{{\partial q_{r} }}{\partial y} + \frac{{\hat{\mu }_{hnf} }}{{\left( {\hat{\rho }C_{p} } \right)_{hnf} }}\left( {\frac{{\partial \omega_{\,1} }}{\partial y}} \right)^{2} \\ & \quad - \frac{{H_{0} }}{{\left( {\hat{\rho }C_{p} } \right)_{hnf} }}\left( {\theta - \theta_{\infty } } \right) + \frac{{\hat{\sigma }_{hnf} }}{{\left( {\hat{\rho }C_{p} } \right)_{hnf} }}\xi_{0}^{2} \,\sin^{2} \alpha \,\omega_{\,1}^{2} . \end{aligned}$$

The accompanying boundary conditions for the model are given as:

4a $${\text{at}}\,\,y = 0:\,\,\,\,\left\{ \begin{aligned} \omega_{\,1} & = \lambda_{1} \left( x \right) + C\frac{{\hat{\mu }_{hnf} }}{{\hat{\rho }_{hnf} }}\frac{{\partial \omega_{\,1} }}{\partial y},\,\, \hfill \\ \,\omega_{\,2} & = \lambda_{2} \left( x \right),\,\,\theta = \theta_{\varepsilon } \left( x \right) + \delta \frac{\partial \theta }{{\partial y}}, \hfill \\ \end{aligned} \right.$$

4b $${\text{as}}\,\,y \to \infty{:}\,\,\omega_{\,1} \to 0\,\,{\text{and}}\,\,\theta \to \theta_{\infty } .$$

In aforesaid equations, $\omega_{\,1} \,{\text{and}}\,\omega_{\,2}$ describe the hybrid nanofluid velocities along *x* and *y* axes respectively, $\theta$ indicates the hybrid nanofluid temperature, $\lambda_{1} \left( x \right) = \lambda_{0} e^{x/l}$ denotes stretchable sheet velocity, $\theta_{\varepsilon } \left( x \right) = \theta_{\infty } + \left( {\theta_{0} - \theta_{\infty } } \right)e^{ax/2l}$ reflects the hybrid nanofluid exponential temperature distribution over the sheet. $a$, $\theta_{0}$ and $\lambda_{0}$ are respectively the temperature distribution parameter in the stretchable sheet, temperature and velocity references, $C = C_{1} /e^{x/2l}$ signifies the Navier’s velocity slip factor in which $C_{1}$ shows the primary slip velocity and $\delta = \delta_{1} /e^{x/2l}$ indicates thermal slip factor wherein $\delta_{1}$ is preliminary thermal slip value. Both the thermal and velocity slip factors got changed owing to the coordinate variable $x$ and no-slip takes place when $\delta = C = 0$. $H_{0}$ represents the coefficient of heat absorption. The blowing/suction velocity is mentioned by $\lambda_{2} \left( x \right) = v_{0} e^{x/2l}$ where $v_{0}$ is the primary blowing/suction velocity strength.

For the developed hybrid nanofluid, the heat capacity, dynamic viscosity, density, thermal conductivity and electrical conductivity are denoted by $\left( {\hat{\rho }C_{p} } \right)_{hnf} ,\,\,\hat{\mu }_{hnf} ,\,\,\hat{\rho }_{hnf} ,\,\,\,\hat{K}_{hnf}$ and $\hat{\sigma }_{hnf}$ respectively and are defined by the relations as mentioned in Table 1. Further, in the Table 1, the volume fraction of graphene and MoS_2_ nanoparticles are respectively signified by $\phi_{\,1} \,{\text{and}}\,\,\phi_{\,2} .$ $\hat{\mu }_{f}$ represents the dynamic viscosity of ethylene glycol, $\hat{\rho }_{g1} \,{\text{and}}\,\hat{\rho }_{m2}$ signify the density of graphene and MoS_2_ nanoparticles, $\hat{\sigma }_{f}$ is used to represent the electrical conductivity of ethylene glycol, $\hat{\sigma }_{g1}$ divulges the electrical conductivity of graphene, $\hat{\sigma }_{m2}$ embodies the electrical conductivity of MoS_2_. $\left( {\hat{\rho }C_{p} } \right)_{f} ,\,\,\left( {\hat{\rho }C_{p} } \right)_{g1} {\text{and}}\,\,\left( {\hat{\rho }C_{p} } \right)_{m2}$ indicate the heat capacitance whereas $\hat{k}_{f} ,\,\,\hat{k}_{g1} \,{\text{and}}\,\hat{k}_{m2}$ represents the thermal conductivity of ethylene glycol, graphene and MoS_2_ respectively. The physical properties of ethylene glycol, graphene and MoS_2_ are reported in Table 2.

**Table 1:** Thermophysical properties and relations of hybrid nanofluids^56,57^.

Properties	Hybrid nanofluid
Heat capacity	$\left( {\hat{\rho }C_{p} } \right)_{hnf} = \left\{ {\left( {1 - \phi_{\,1} } \right)\left( {\hat{\rho }C_{p} } \right)_{f} + \phi_{\,1} \left( {\hat{\rho }C_{p} } \right)_{g1} } \right\}\left( {1 - \phi_{\,1} } \right) + \phi_{\,2} \left( {\hat{\rho }C_{p} } \right)_{m2}$
Dynamic viscosity	$\hat{\mu }_{hnf} = \hat{\mu }_{f} \left\{ {\left( {1 - \phi_{1} } \right)\left( {1 - \phi_{2} } \right)} \right\}^{ - 2.5}$
Density	$\hat{\rho }_{hnf} = \left\{ {1 + \phi_{\,1} \left( {\hat{\rho }_{g1} - \hat{\rho }_{f} } \right)} \right\}\left( {1 - \phi_{\,2} } \right) + \phi_{\,2} \hat{\rho }_{m2}$
Thermal conductivity	$\hat{K}_{hnf} = \left\{ {\frac{{2\hat{k}_{nf} + \hat{k}_{m2} + 2\phi_{2} \left( {\hat{k}_{m2} - \hat{k}_{nf} } \right)}}{{2\hat{k}_{nf} + \hat{k}_{m2} - \phi_{2} \left( {\hat{k}_{m2} - \hat{k}_{nf} } \right)}}} \right\}\hat{k}_{nf}$, where $\hat{k}_{nf} = \left\{ {\frac{{2\hat{k}_{f} + \hat{k}_{g1} + 2\phi_{\,1} \left( {\hat{k}_{g1} - \hat{k}_{f} } \right)}}{{2\hat{k}_{f} + \hat{k}_{g1} - \phi_{1} \left( {\hat{k}_{g1} - \hat{k}_{f} } \right)}}} \right\}\hat{k}_{f}$
Electrical conductivity	$\hat{\sigma }_{hnf} = \left\{ {\frac{{2\phi_{\,2} \left( {\hat{\sigma }_{m2} - \hat{\sigma }_{nf} } \right) + 2\hat{\sigma }_{nf} + \hat{\sigma }_{m2} }}{{\phi_{\,2} \left( {\hat{\sigma }_{nf} - \hat{\sigma }_{m2} } \right) + 2\hat{\sigma }_{nf} + \hat{\sigma }_{m2} }}} \right\}\hat{\sigma }_{nf} ,$ where $\hat{\sigma }_{nf} = \hat{\sigma }_{f} \left\{ {\frac{{3\phi_{\,1} \left( {\hat{\sigma } - 1} \right)}}{{\left( {1 - \phi_{\,1} } \right)\hat{\sigma } + \left( {2 + \phi_{\,1} } \right)}} + 1} \right\}\,\,{\text{and}}\,\,\hat{\sigma } = \frac{{\hat{\sigma }_{g1} }}{{\hat{\sigma }_{f} }}\,$

**Table 2:** The physical properties of ethylene glycol, graphene and MoS_2_^2,35^.

	Ethylene glycol	Graphene	MoS_2_
$\hat{\rho } \; \left( {{\text{kg/m}}^{{3}} } \right)$	1115	2250	5060
$\hat{k} \; \left( {{\text{W/m}}\,{\text{K}}} \right)$	0.253	2500	904.4
$\hat{\sigma } \; \left( {\text{S/m}} \right)$	$1.10 \times 10^{ - 4}$	$1 \times 10^{7}$	$2.09 \times 10^{4}$
$C_{p} \; \left( {{\text{J/kg}}\,{\text{K}}} \right)$	2430	2100	397.21
$\phi$	0.20	0.025	0.030

Following the works of Hussanan et al.^58^ and Brewster^59^, an optically thick fluid has been considered here, the radiation heat flux $q_{r}$ with the help of Rosseland approximation can be expressed as:

5 $$q_{r} = - \frac{{4\sigma^{ * } }}{{3k^{ * } }}\frac{{\partial \theta^{4} }}{\partial y}.$$

In above expression (5), $\sigma^{*}$ signifies the Stefan-Boltzmann constant while $k^{ * }$ specifies the heat absorption constant. Further, to linearize the term $\theta^{4}$ the Taylor’s series is implemented to expand it about $\theta_{\infty }$(free stream temperature) and is expressed after ignoring higher degree terms as under:

6 $$\theta^{4} \cong \,\theta_{\infty }^{3} \left( {4\theta - 3\theta_{\infty } } \right).$$

The energy Eq. (3) with the help of Eqs. (5) and (6) is reduced to

7 $$\begin{aligned} \omega_{\,1} \frac{\partial \theta }{{\partial x}} + \omega_{\,2} \frac{\partial \theta }{{\partial y}} & = \left\{ {\frac{{\hat{K}_{hnf} }}{{\left( {\hat{\rho }C_{p} } \right)_{hnf} }} + \frac{{16T_{\infty }^{3} \sigma^{ * } }}{{3\left( {\hat{\rho }C_{p} } \right)_{hnf} k^{ * } }}} \right\}\frac{{\partial^{2} \theta }}{{\partial y^{2} }} + \frac{{\hat{\mu }_{hnf} }}{{\left( {\hat{\rho }C_{p} } \right)_{hnf} }}\left( {\frac{{\partial \omega_{\,1} }}{\partial y}} \right)^{2} \hfill \\ & \quad - \frac{{H_{0} }}{{\left( {\hat{\rho }C_{p} } \right)_{hnf} }}\left( {\theta - \theta_{\infty } } \right) + \frac{{\hat{\sigma }_{hnf} }}{{\left( {\hat{\rho }C_{p} } \right)_{hnf} }}\xi_{0}^{2} \sin^{2} \sigma \,\omega_{\,1}^{2} . \hfill \\ \end{aligned}$$

## Numerical solution

The mathematical model reported in the above section includes highly nonlinear partial differential equations, therefore, to identify the numerical solution of these equations subject to the allied conditions, it is relevant to introduce a stream function $\psi$ and similarity variables $\eta$ as under^60^:

8 $$\omega_{\,1} = \frac{\partial \psi }{{\partial y}},\,\,\omega_{\,2} = - \frac{\partial \psi }{{\partial x}},$$

9 $$\left. \begin{aligned} \eta \left( {x,\,y} \right) & = \sqrt {\text{Re}} \,e^{x/2l} \left( {\frac{y}{\sqrt 2 l}} \right),\,\,\psi \left( {x,\,y} \right) = \upsilon_{f} \,e^{x/2l} \sqrt {2{\text{Re}} } \,\xi \left( \eta \right),\, \hfill \\ \,\theta \left( {x,\,y} \right) & = \theta_{\infty } - \left( {\theta_{\infty } - \theta_{0} } \right)e^{ax/2l} T\left( \eta \right), \hfill \\ \end{aligned} \right\}$$

where, ${\text{Re}} ,\,\,\upsilon_{f} \,\,{\text{and}}\,\,T\left( \eta \right)$ signify the Reynolds number, kinematic viscosity of water and hybrid nanofluid temperature in dimensionless form respectively.

The Eqs. (8) and (9) result the following relations

10 $$\omega_{\,1} = \frac{{\upsilon_{f} {\text{Re}} }}{l}e^{x/l} \xi^{\prime}\left( \eta \right)\,\,{\text{and}}\,\,\,\omega_{\,2} = \frac{{ - \upsilon_{f} \sqrt {2{\text{Re}} } }}{l}\left\{ {\eta \xi^{\prime}\left( \eta \right) + \xi \left( \eta \right)} \right\}e^{x/2l} .$$

Here, prime shows the differentiation with regard to similarity variable $\eta$. Further, Eqs. (9) and (10) transform the mathematical model reported in “Mathematical foundation of the problem” section in below mentioned dimensionless forms:

11 $$\frac{{\hat{\mu }_{hnf} /\hat{\mu }_{f} }}{{\hat{\rho }_{hnf} /\hat{\rho }_{f} }}\xi^{\prime\prime\prime}\left( \eta \right) + \xi^{\prime\prime}\left( \eta \right)\xi \left( \eta \right) - \left\{ {\xi^{\prime}\left( \eta \right)} \right\}^{2} - \frac{{\hat{\sigma }_{hnf} /\hat{\sigma }_{f} }}{{\hat{\rho }_{hnf} /\hat{\rho }_{f} }}2M\,e^{ - X} \sin^{2} \alpha \,\xi^{\prime}\left( \eta \right) = 0,$$

12 $$\begin{aligned} \frac{1}{\Pr }\left\{ {\frac{{\hat{K}_{hnf} }}{{\hat{k}_{f} }} + Tr} \right\}T^{\prime\prime}\left( \eta \right) + \left\{ {\frac{{\hat{\mu }_{hnf} }}{{\hat{\mu }_{f} }}e^{X} \xi^{\prime\prime}\left( \eta \right)^{2} + \frac{{\hat{\sigma }_{hnf} }}{{\hat{\sigma }_{f} }}2\,M\,\sin^{2} \alpha \,\xi^{\prime}\left( \eta \right)^{2} } \right\}Ec\,e^{{\left( {2 - a} \right)X}} \hfill \\ \quad \quad - \,\,\frac{{\left( {\hat{\rho }C_{p} } \right)_{hnf} }}{{\left( {\hat{\rho }C_{p} } \right)_{f} }}\left\{ {aT\left( \eta \right)\xi^{\prime}\left( \eta \right) - \xi \left( \eta \right)T^{\prime}\left( \eta \right)} \right\} = 2H_{a} \,e^{ - X} T\left( \eta \right), \hfill \\ \end{aligned}$$

with allied boundary conditions

13 $$\left. \begin{aligned} & \xi^{\prime}\left( \eta \right) = L\,\xi^{\prime\prime}\left( \eta \right) + 1,\,\,\xi \left( \eta \right) = S\,\,{\text{and}}\,\,T\left( \eta \right) = D\,T^{\prime}\left( \eta \right) + 1\,\,\,{\text{at}}\,\,\eta = 0, \hfill \\ & \xi^{\prime}\left( \eta \right) \to 0\,\,\,{\text{and}}\,\,T\left( \eta \right)\,\,{\text{as}}\,\eta \to \infty . \hfill \\ \end{aligned} \right\}$$

The dimensionless parameters reported in the Eqs. (11) to (13) are $M,\,\,X,\,\,Tr,\,\,\Pr ,\,\,{\text{Re}} ,\,$ $Ec,\,\,L,\,\,H_{a} ,\,\,S\,\,{\text{and}}\,\,D$ which represent the magnetic parameter, dimensionless coordinate, radiation parameter, Prandtl number, Reynolds number, Eckert number, velocity slip, heat absorption, injection/suction and thermal slip parameters respectively. These parameters are defined by below mentioned relations:

14 $$\left. \begin{aligned} X &= \frac{x}{l},\,\,M = \frac{{\left( {\xi_{0} l} \right)^{2} \hat{\sigma }_{f} }}{{\hat{\mu }_{f} {\text{Re}} }},\,\,Tr = \frac{16}{3}\frac{{T_{\infty }^{3} \sigma^{*} }}{{\hat{k}_{f} k^{*} }},\,\,\Pr = \frac{{\left( {\hat{\rho }C_{p} \upsilon } \right)_{f} }}{{\hat{k}_{f} }},\,\, \hfill \\ {\text{Re}} & = \frac{{l\lambda_{0} }}{{\upsilon_{f} }},\,Ec = \frac{{\lambda_{0}^{2} }}{{\left( {C_{p} } \right)_{f} \left( {T_{0} - T_{\infty } } \right)}},\,\,L = \frac{{\hat{\mu }_{hnf} }}{{\hat{\rho }_{hnf} }}\frac{{C_{1} }}{l}\sqrt {\frac{{\text{Re}}}{2}} ,\, \hfill \\ H_{a} & = \frac{{H_{0} l^{2} }}{{\left( {\hat{\rho }C_{p} } \right)_{f} \upsilon_{f} {\text{Re}} }},\,\,S = \,\frac{{lv_{0} }}{{\upsilon_{f} }}\sqrt {\frac{2}{{\text{Re}}}} \,\,{\text{and}}\,\,D = \frac{{\delta_{1} }}{l}\sqrt {\frac{{\text{Re}}}{2}} . \hfill \\ \end{aligned} \right\}$$

### Wall temperature gradient and skin friction coefficients

To scrutinize the wall heat transport rate and shear stress function from the engineering outlooks, the expressions of local Nusselt number $Nn_{x}$ and skin friction coefficients $Sf_{x}$ are derived in both dimension and dimensionless forms. The dimension forms of $Nn_{x}$ and $Sf_{x}$ are defined as:

15 $$Nn_{x} = \frac{{x\tau_{w} }}{{\hat{k}_{f} \left( {\theta_{\varepsilon } - \theta_{\infty } } \right)}}\;{\text{and}}\;Sf_{x} = \frac{{\xi_{w} }}{{\lambda_{1}^{2} \rho_{f} }},$$

where $\tau_{w} = - \hat{K}_{hnf} \left( {\frac{\partial \theta }{{\partial y}}} \right)_{y = 0} + \left( {q_{r} } \right)_{y = 0}$ and $\xi_{w} = \hat{\mu }_{hnf} \left( {\frac{{\partial \omega_{\,1} }}{\partial y}} \right)_{y = 0}$ are respectively the wall heat flux and wall shear stress. The dimensionless forms of $Nn_{x}$ and $Sf_{x}$ are expressed as under:

16 $$\left. \begin{aligned} & Nn_{x} = - \left( {\frac{{\hat{K}_{hnf} }}{{\hat{k}_{f} }} + Tr} \right)\sqrt {\frac{{X{\text{Re}}_{x} }}{2}} T^{\prime}\left( 0 \right)\,\, \hfill \\ & {\text{and}}\,\,Sf_{x} = \frac{{\hat{\mu }_{hnf} }}{{\hat{\mu }_{f} }}\frac{1}{{\sqrt {2{\text{Re}}_{x} } }}\xi^{\prime\prime}\left( 0 \right), \hfill \\ \end{aligned} \right\}$$

where ${\text{Re}}_{x} = \lambda_{1} \left( x \right)x/\upsilon_{f}$ signifies the local Reynolds number.

### Numerical technique implementation

To illustrate physically consistent and stable numerical solution corresponding to transformed similarity Eqs. (11) and (12) satisfying the allied conditions (13) the finite difference technique based bvp4c solver present in MATLAB is implemented. This solver was originally developed by Kierzenka and Shamoine^61^ which works on three stages Lobatto IIIa algorithm. The algorithm results in 4th order accurate and uniform solution within the given range. The involved steps of the aforesaid method are furnished below:

Step 1: Foremost, the new variables are introduced for the transformed similarity Eqs. (11) and (12) as under:
  17 $$\left. \begin{aligned} & y\left( 1 \right) = \xi \left( \eta \right),\,\,y\left( 2 \right) = \xi^{\prime}\left( \eta \right),\,\,y\left( 3 \right) = \xi^{\prime\prime}\left( \eta \right), \hfill \\ & y\left( 4 \right) = T\left( \eta \right)\,\,{\text{and}}\,\,y\left( 5 \right) = T^{\prime}\left( \eta \right). \hfill \\ \end{aligned} \right\}$$
Step 2: Then, by making use of relations (17), Eqs. (11) and (12) are changed into system of first order equations:
  18 $$\left. \begin{aligned} \xi^{\prime}\left( \eta \right) & = y\left( 2 \right), \hfill \\ \xi^{\prime\prime}\left( \eta \right) & = y\left( 3 \right), \hfill \\ \xi^{\prime\prime\prime}\left( \eta \right) & = \frac{{\phi_{a} }}{{\phi_{c} }}\left[ {\left\{ {y\left( 2 \right)} \right\}^{2} - y\left( 1 \right)y\left( 3 \right) + \frac{{2\phi_{b} }}{{\phi_{a} }}M\,e^{ - X} \sin^{2} \alpha \,y\left( 2 \right)} \right], \hfill \\ T^{\prime}\left( \eta \right) & = y\left( 5 \right), \hfill \\ T^{\prime\prime}\left( \eta \right) & = \frac{\Pr }{{\left( {\phi_{e} + Tr} \right)}}\left[ {\phi_{d} \left\{ {ay\left( 4 \right)y\left( 2 \right) - y\left( 1 \right)y\left( 5 \right)} \right\} - } \right. \hfill \\ \,\,\,\,\,\,\,\,\,\,\,\,\,\,\,\,\,\,\, & \quad \left. {\left\{ {2\phi_{b} M\,\sin^{2} \alpha \,\left( {y\left( 2 \right)} \right)^{2} + \phi_{c} e^{X} \left( {y\left( 3 \right)} \right)^{2} } \right\}Ec\,e^{{\left( {2 - a} \right)X}} + 2e^{ - X} H_{a} \,y\left( 4 \right)} \right], \hfill \\ \end{aligned} \right\}$$
  where, $$\phi_{a} = \frac{{\hat{\rho }_{hnf} }}{{\hat{\rho }_{f} }},\,\,\phi_{b} = \frac{{\hat{\sigma }_{hnf} }}{{\hat{\sigma }_{f} }},\,\,\phi_{c} = \frac{{\hat{\mu }_{hnf} }}{{\hat{\mu }_{f} }},\,\,\phi_{d} = \frac{{\left( {\hat{\rho }C_{p} } \right)_{hnf} }}{{\left( {\hat{\rho }C_{p} } \right)_{f} }}\,\,{\text{and}}\,\,\phi_{e} = \frac{{\hat{K}_{hnf} }}{{\hat{k}_{f} }}$$.

Step 3: According to the assumed variables (18), the boundary conditions (13) are expressed as:
  19 $$\left. \begin{aligned} y_{a} \left( 2 \right) & = 1 - L\,y_{a} \left( 3 \right),\,\,y_{a} \left( 1 \right) = S,\,\,y_{a} \left( 4 \right) = 1 + D\,y_{a} \left( 5 \right),\, \hfill \\ y_{b} \left( 2 \right) & = 0\,\,{\text{and}}\,\,y_{b} \left( 4 \right) = 0. \hfill \\ \end{aligned} \right\}$$
  Here, subscript *a* indicates the initial sheet position i.e., $\eta = 0$ while subscript *b* represents the boundary condition at infinity. The value of $\eta$ is considered as $\eta = 5$ for the infinite boundary condition.
Step 4: Finally, the initial guess was provided at the initial mesh points to obtain the solution. These steps are repeated till the obtained numerical solutions satisfy the boundary conditions (19) asymptotically.

### Validation of numerical findings and scheme

The obtained results and the employed numerical technique have been validated by comparing the numerical entities of $Nn_{x}$(local Nusselt number) with those of Wahid et al.^37^ and have reported in tabular form. In pursuance, foremost the values of the magnetic parameter, aligned magnetic field angle, radiation parameter and Eckert number are respectively taken as $M = 0,\,\,\alpha = 0,$ $Tr = 0$ and $Ec = 0$ to convert the mathematical model comparable to Wahid et al.^37^. Further, the values of $Nn_{x}$ are computed for the varying values of two different flow parameters *S* and *D* with the developed scheme of three stages Lobatto IIIa-bvp4c solver by considering water-Cu-Al_2_O_3_ hybrid nanofluid (as chosen by Wahid et al.^37^) instead of ethylene glycol-based hybrid nanofluid containing graphene and MoS_2_ nanoparticles. These computed values are presented in Table 3, which disclose venerable agreement between the numerical results and executed numerical scheme. Thus, it reveals that the developed numerical scheme and the results of this paper are acceptable and valid.

**Table 3:** Comparison of obtained numerical entities of $- Nn_{x}$ with existing results of Wahid et al.^37^.

$S$	$D$	$- Nn_{x}$ obtained by Wahid et al.^37^	$- Nn_{x}$ obtained in present study
0.2	0.4	1.459270	1.459271
0.3	0.4	1.513239	1.513242
0.4	0.4	1.565144	1.565149
0.3	0.2	2.156547	2.156546
0.3	0.4	1.165551	1.165554
0.3	0.6	1.513240	1.513242

## Results and discussion

**Figure 2:**
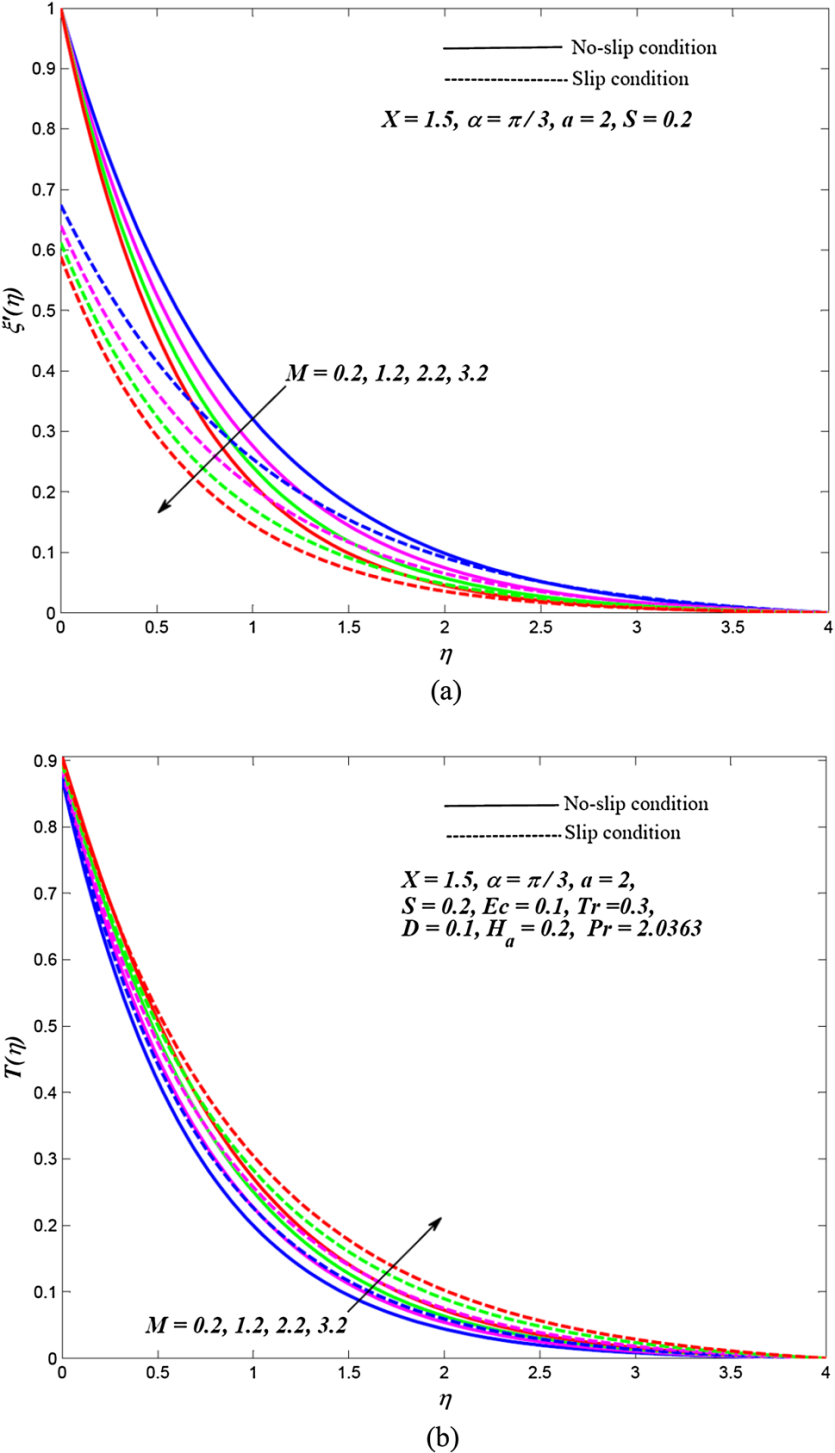
Effects of *M* on the dispersal profiles of hybrid (**a**) velocity $\xi^{\prime}\left( \eta \right)$ and (**b**) temperature $T\left( \eta \right)$.

**Figure 3:**
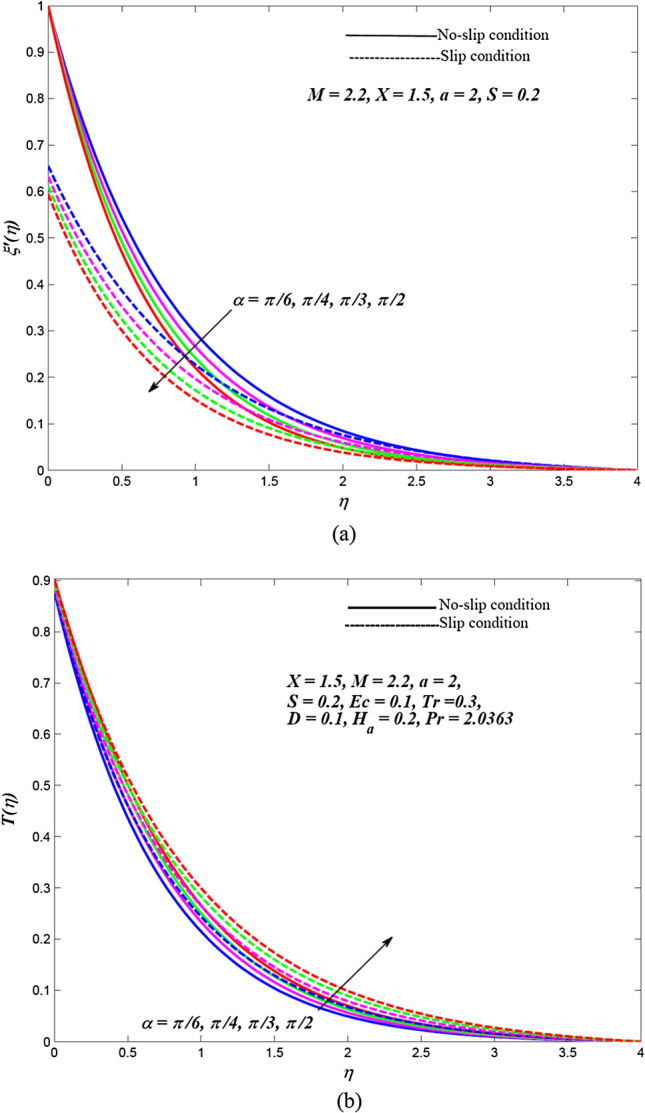
Effects of $\alpha$ on the dispersal profiles of hybrid (**a**) velocity $\xi^{\prime}\left( \eta \right)$ and (**b**) temperature $T\left( \eta \right)$.

**Figure 4:**
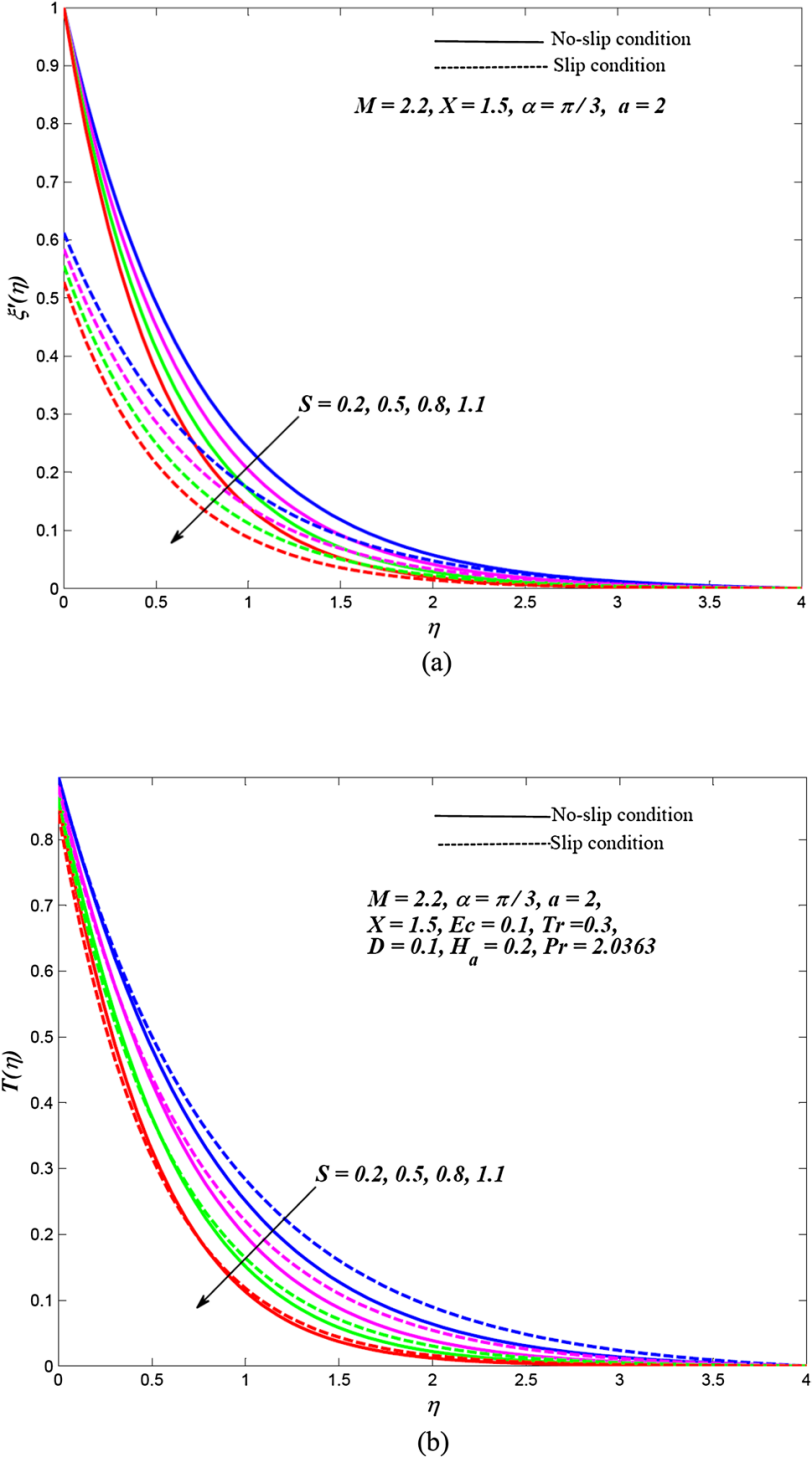
Effects of $S\,\left( { > 0} \right)$ on the dispersal profiles of hybrid (**a**) velocity $\xi^{\prime}\left( \eta \right)$ and (**b**) temperature $T\left( \eta \right)$.

**Figure 5:**
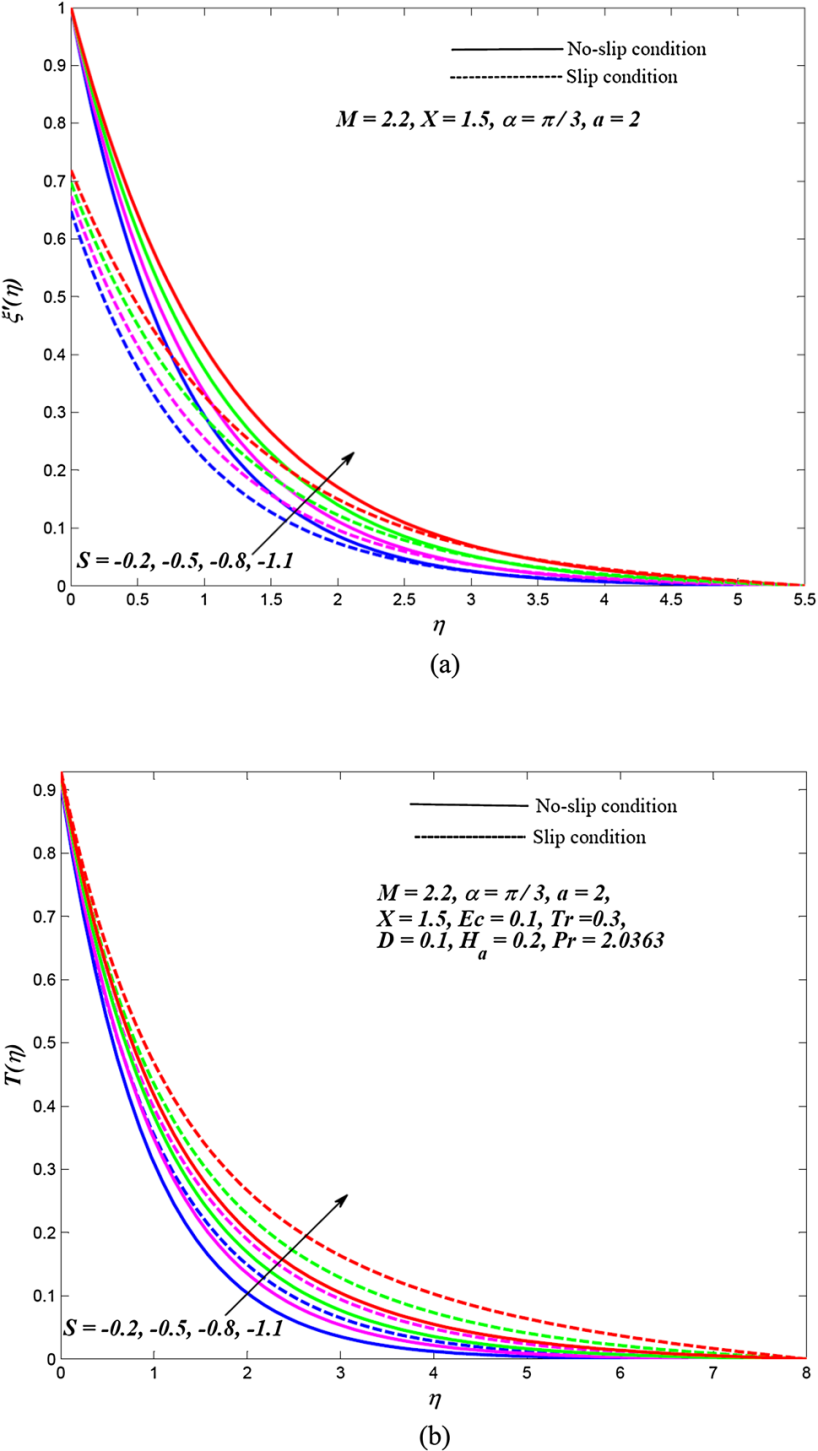
Effects of $S\,\left( { < 0} \right)$ on the dispersal profiles of hybrid (**a**) velocity $\xi^{\prime}\left( \eta \right)$ and (**b**) temperature $T\left( \eta \right)$.

**Figure 6:**
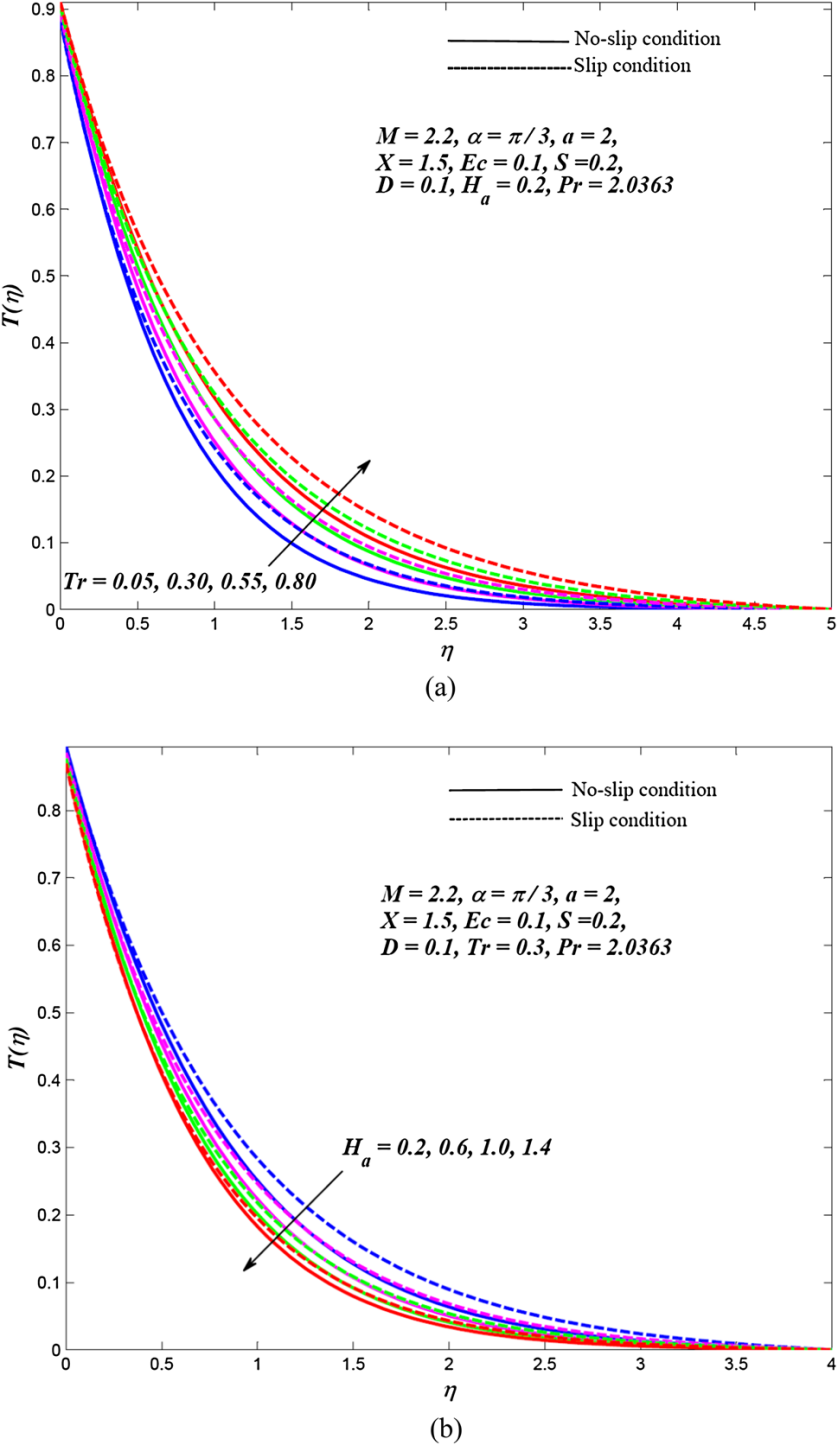
Hybrid temperature dispersal profiles $T\left( \eta \right)$ for varying (**a**) $Tr$ and (**b**) $H_{a}$.

**Figure 7:**
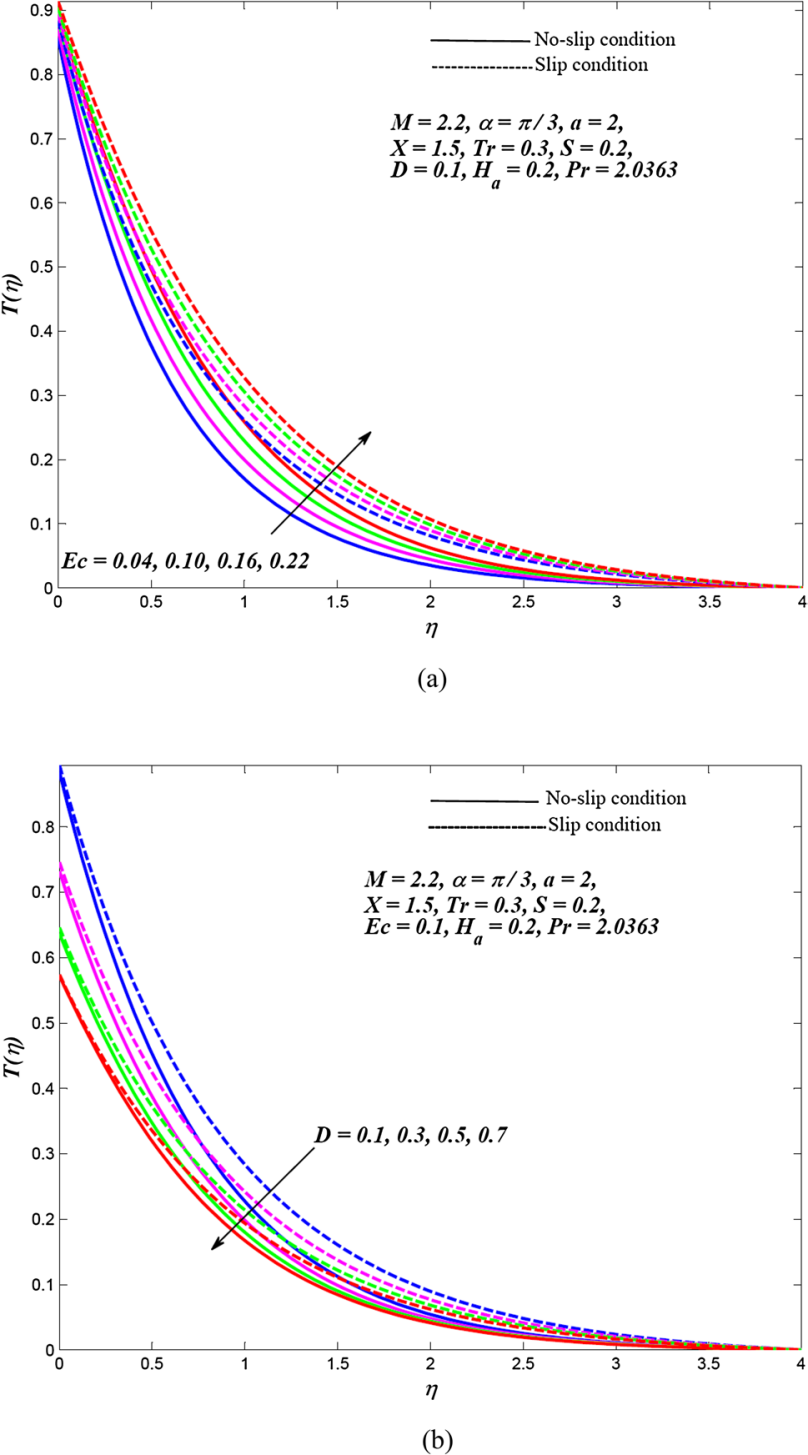
Hybrid temperature dispersal profiles $T\left( \eta \right)$ for varying (**a**) $Ec$ and (**b**) $D$.

Due to the enormously nonlinear nature and intricacy, the solution of leading Eqs. (11) and (12) subjected to allied boundary conditions (13) are numerically solved utilizing three-stages Lobatto-IIIa-bvp4c solver based on a finite difference scheme in MATLAB. The involved steps of the implemented method are mentioned in the previous subsection. In this section, the effects of emerging physical parameters on the dynamics of ethylene glycol-based hybrid nanofluid flow containing graphene and MoS_2_ nanoparticles over an exponentially stretchable sheet with and without velocity slip conditions are captured through graphical results and tables. Also, a physical description of the behaviour of influencing flow parameters is provided. In the depicted graphical results, solid lines indicate the significance of inducing flow parameters under no-slip velocity conditions, while dashed lines signify the influence in the case of velocity slip conditions. For the numerical computation, the default values of temperature distribution parameter and Prandtl number are respectively fixed as $a = 2,\,\,X = 1.5$ and $\Pr = 2.0363$(for ethylene glycol base fluid) while other regulatory flow parameters such as magnetic parameter (*M*), angle of aligned magnetic field $(\alpha )$, Eckert number (*Ec*), injection/suction (*S*), heat absorption parameter (*H*_*a*_), velocity slip parameter (*L*), radiation parameter (*Tr*) and thermal slip parameter (*D*) are considered to be varied to capture their significance on the flow field and heat transfer features. The bearings of the magnetic field, the angle of aligned magnetic field, injection/suction, thermal radiation, heat absorption, Joule dissipation and thermal slip parameter on the velocity and temperature dispersal profiles of ethylene glycol-graphene-MoS_2_ conveying hybrid nanofluid have been portrayed in Figs. 2, 3, 4, 5, 6, 7 for both velocity slip and without slip conditions. It is elucidated from Fig. 2 that for both velocity slip $(L = 0.5)$ and no-slip $(L = 0)$ situations, the hybrid nanofluid velocity $f^{\prime}(\eta )$ is reduced while the temperature of hybrid nanofluid $T(\eta )$ is raised with the gradual augmentation of strength of magnetic field. This phenomenon happens because a resisting force is persuaded due to the gradual improvement in the strength of the magnetic field. This resisting force termed as Lorentz force acts in the opposite direction of flow-field as well as hybrid nanoparticles and subsequently, the velocity of hybrid nanofluid is retarded whereas its temperature gets improved due to enrichment in frictional drag force. The hybrid nanofluid velocity profiles are declined while the temperature dispersal profiles are improved owing to the rise in the angle of aligned magnetic field under both velocity slip and without slip conditions, as revealed in Fig. 3. This tendency of profiles exhibits that the angle of the aligned magnetic field retards the velocity of hybrid nanofluid and the resistive force’s strength is optimal when the exerted magnetic field is perpendicular to the stretchable sheet. The optimal resistive force diminishes the motion hybrid nanofluid and improves the temperature of the fluid. Figures 4 and 5 represent the diminishing effect of suction parameter $(S > 0)$ on the scattering profiles of $f^{\prime}(\eta )\,\,{\text{and}}\,\,T(\eta )$ while these profiles are reversed in case of the injection parameter ($S < 0$). The reason behind this behaviour of profiles is that the momentum boundary layer sticks to the surface of the stretchable sheet in the instance of suction, which breaks the flow momentum and as a result, both velocity and temperature of hybrid nanofluid are reduced. On the other hand, injection enhances fluid via lateral mass flux over the stretchable sheet and, in turn, appends the momentum of fluid flow. Consequently, both the velocity and temperature of hybrid nanofluid get improved. Figure 6 illustrates the influence of thermal radiation and heat absorption on the temperature of hybrid nanofluid. This figure shows that the temperature dispersal profiles upsurge due to the rise in the values of the radiation parameter (*Tr*). Generally, thermal radiation depends on the temperature of the surrounding, and it is emitted in an electromagnetic waveform. Therefore, thermal radiation can be assumed as a function of temperature. The thermal effect improves the conduction properties of nanofluid and hence, thicken the boundary layer and as a result, the temperature of fluid gets enhanced. Further, the temperature dispersal profiles of hybrid nanofluid are reduced due to the enhancement in heat absorption parameter (*H*_*a*_). The reason behind this physical behaviour of the fluid is that a rise in the values of *H*_*a*_ results from the augmentation in the heat-absorbing capacity of the hybrid nanofluid and consequently, the fluid temperature gets diminished. Figure 7 is depicted to capture the inspirations of viscous dissipation and thermal slip factor on the temperature of hybrid nanofluid. It is observed that the temperature dispersal profiles of hybrid nanofluid are improved owing to the enhancement in Eckert number (*Ec*) while it reduced due to thermal slip factor (*D*). Since, Eckert number ($Ec$) represents the relation between enthalpy and kinetic energy. It is involved in converting kinetic energy to the form of internal energy in opposing the viscous fluid stresses. As, $Ec$ increases the internal energy also gets increased and consequently fluid temperature gets improved. This infers that the inspiration of viscous dissipation is responsible to raise the temperature of hybrid nanofluid. On the other hand, the thermal slip parameter is accountable to lessen the hybrid nanofluid temperature under both the velocity slip and no-slip situations. Physically, it is justified because from the surface of the stretchable sheet a reduced quantity of heat flows to the hybridized fluid in increasing the thermal slip parameter. Additionally, the graphical illustrations suggest that the thermal boundary layer thickness is improved by the magnetic field, angle of aligned magnetic field, viscous dissipation, injection and thermal radiation while it is declined due to augmentation in suction, heat absorption, and the thermal slip factor.

The wall heat transport rate and shear stress function are analysed from the engineering outlooks. For this, the numerical form of local Nusselt number $Nn_{x}$ and skin friction coefficients $Sf_{x}$ are obtained under both the velocity slip $\left( {L = 0.5} \right)$ and no-slip $\left( {L = 0} \right)$ situations against emerging flow parameters. These values are provided in Tables 4 and 5. It is perceived from Table 4 that the numerical values of $Sf_{x}$ are increasing owing to rise in magnetic field, angle of aligned magnetic field and suction parameter $\left( {S > 0} \right)$ whereas these values are decreased due to injection parameter $\left( {S < 0} \right)$. This unveils that at the surface of the stretchable sheet under both velocity slip and no-slip situations the shear stress function is improved owing to upsurge in the angle of aligned magnetic field, suction and magnetic field while it gets reduced due to injection. Table 5 illustrates that under both the situations of velocity slip and no-slip, the numerical findings of $Nn_{x}$ are reduced by increasing angle of aligned magnetic field, Eckert number, magnetic, injection and thermal slip parameters whereas these numeric are enhanced due to upsurge in heat absorption, suction and radiation parameters. This quantifies that the heat transport rate can be improved by the inspirations of heat absorption, suction and thermal radiation while viscous dissipation, magnetic field, injection, angle of aligned magnetic field and thermal slip factor are significant to reduce the heat transport rate of hybrid nanofluid. Further, it is noticed that the shear stress function at the surface of the stretchable sheet is lower in the case of velocity slip condition as compared to the no-slip condition.

**Table 4:** Numerical findings of $Sf_{x}$ with velocity slip $\left( {L = 0.5} \right)$ and without slip $\left( {L = 0} \right)$ conditions.

$M$	$\alpha$	$S\left( { > 0} \right)$ Suction	$S\left( { < 0} \right)$ Injection	$- Sf_{x}$(skin friction coefficient)	
				$L = 0$	$L = 0.5$
**0.2**	$\pi /3$	0.2	− 0.2	0.984548	0.566347
**1.2**	$\pi /3$	0.2	− 0.2	1.117289	0.626290
**2.2**	$\pi /3$	0.2	− 0.2	1.235613	0.674850
**3.2**	$\pi /3$	0.2	− 0.2	1.343110	0.715361
2.2	$\varvec {\pi} /\bf{6}$	0.2	− 0.2	1.057414	0.599995
2.2	$\varvec {\pi}/\bf{4}$	0.2	− 0.2	1.150075	0.640188
2.2	$\varvec {\pi}/\bf{3}$	0.2	− 0.2	1.235613	0.674850
2.2	$\varvec {\pi}/\bf{2}$	0.2	− 0.2	1.315349	0.705207
2.2	$\pi /3$	**0.2**	− 0.2	1.235613	0.674850
2.2	$\pi /3$	**0.5**	− 0.2	1.380478	0.723757
2.2	$\pi /3$	**0.8**	− 0.2	1.538953	0.773212
2.2	$\pi /3$	**1.1**	− 0.2	1.710059	0.822208
2.2	$\pi /3$	0.2	**− 0.2**	1.235613	1.064193
2.2	$\pi /3$	0.2	**− 0.5**	1.380478	0.952448
2.2	$\pi /3$	0.2	**− 0.8**	1.538953	0.854313
2.2	$\pi /3$	0.2	**− 1.1**	1.710059	0.768793

The varying values of influencing parameters are highlighted with bold numbers.

**Table 5:** Numerical findings of $Nn_{x}$ with velocity slip $\left( {L = 0.5} \right)$ and without slip $\left( {L = 0} \right)$ conditions.

$M$	$\alpha$	$S\left( { > 0} \right)$ Suction	$S\left( { < 0} \right)$ Injection	$Tr$	$H_{a}$	$Ec$	$D$	$- Nn_{x}$(Nusselt number)	
								$L = 0$	$L = 0.5$
**0.2**	$\pi /3$	0.2	− 0.2	0.30	0.2	0.10	0.1	1.644858	1.558717
**1.2**	$\pi /3$	0.2	− 0.2	0.30	0.2	0.10	0.1	1.485001	1.440140
**2.2**	$\pi /3$	0.2	− 0.2	0.30	0.2	0.10	0.1	1.341105	1.344997
**3.2**	$\pi /3$	0.2	− 0.2	0.30	0.2	0.10	0.1	1.209569	1.266830
2.2	$\varvec {\pi}/\bf{6}$	0.2	− 0.2	0.30	0.2	0.10	0.1	1.557357	1.492048
2.2	$\varvec {\pi}/\bf{4}$	0.2	− 0.2	0.30	0.2	0.10	0.1	1.445241	1.412804
2.2	$\varvec {\pi}/\bf{3}$	0.2	− 0.2	0.30	0.2	0.10	0.1	1.341105	1.344997
2.2	$\varvec {\pi}/\bf{2}$	0.2	− 0.2	0.30	0.2	0.10	0.1	1.243565	1.286309
2.2	$\pi /3$	**0.2**	− 0.2	0.30	0.2	0.10	0.1	1.341105	1.344997
2.2	$\pi /3$	**0.5**	− 0.2	0.30	0.2	0.10	0.1	1.500815	1.559598
2.2	$\pi /3$	**0.8**	− 0.2	0.30	0.2	0.10	0.1	1.675067	1.802270
2.2	$\pi /3$	**1.1**	− 0.2	0.30	0.2	0.10	0.1	1.858951	2.064318
2.2	$\pi /3$	0.2	**− 0.2**	0.30	0.2	0.10	0.1	1.155368	1.107529
2.2	$\pi /3$	0.2	**− 0.5**	0.30	0.2	0.10	0.1	1.041537	0.974620
2.2	$\pi /3$	0.2	**− 0.8**	0.30	0.2	0.10	0.1	0.976806	0.903437
2.2	$\pi /3$	0.2	**− 1.1**	0.30	0.2	0.10	0.1	0.869066	0.791272
2.2	$\pi /3$	0.2	− 0.2	**0.05**	0.2	0.10	0.1	1.214229	1.233974
2.2	$\pi /3$	0.2	− 0.2	**0.30**	0.2	0.10	0.1	1.340256	1.342592
2.2	$\pi /3$	0.2	− 0.2	**0.55**	0.2	0.10	0.1	1.452727	1.438924
2.2	$\pi /3$	0.2	− 0.2	**0.80**	0.2	0.10	0.1	1.554612	1.526204
2.2	$\pi /3$	0.2	− 0.2	0.30	**0.2**	0.10	0.1	1.341105	1.344997
2.2	$\pi /3$	0.2	− 0.2	0.30	**0.6**	0.10	0.1	1.444444	1.465916
2.2	$\pi /3$	0.2	− 0.2	0.30	**1.0**	0.10	0.1	1.537046	1.570846
2.2	$\pi /3$	0.2	− 0.2	0.30	**1.4**	0.10	0.1	1.621544	1.664534
2.2	$\pi /3$	0.2	− 0.2	0.30	0.2	**0.04**	0.1	1.668457	1.470400
2.2	$\pi /3$	0.2	− 0.2	0.30	0.2	**0.10**	0.1	1.341105	1.344997
2.2	$\pi /3$	0.2	− 0.2	0.30	0.2	**0.16**	0.1	1.013742	1.219595
2.2	$\pi /3$	0.2	− 0.2	0.30	0.2	**0.22**	0.1	0.686294	1.094193
2.2	$\pi /3$	0.2	− 0.2	0.30	0.2	0.10	**0.1**	1.341105	1.344997
2.2	$\pi /3$	0.2	− 0.2	0.30	0.2	0.10	**0.3**	1.035557	1.082022
2.2	$\pi /3$	0.2	− 0.2	0.30	0.2	0.10	**0.5**	0.843402	0.905065
2.2	$\pi /3$	0.2	− 0.2	0.30	0.2	0.10	**0.7**	0.711398	0.777853

The varying values of influencing parameters are highlighted with bold numbers.

## Quadratic regression analysis: approximations of skin friction coefficients and wall temperature gradients

In this section, for the quadratic regression approximation, a statistical method is accomplished to know the connection between two or more emerging flow parameters. Precisely, regression approximation is employed to analyse how the features of an emerging flow parameter change due to the variation of another flow parameter while the remaining flow parameters are kept constant. Here, we have presented the quadratic regression approximations analysis on the numerical entities of skin friction coefficients and wall temperature gradients. For the reduced skin friction coefficients $Sf_{x}$, a quadratic regression approximation model is reported for 100 diverse values of suction parameter *S* and velocity slip parameter *L*, chosen randomly within the intervals $\left[ {0.2,\,\,1.1} \right]$ and $\left[ {0,\,\,0.5} \right]$ respectively for enhancing values of the magnetic parameter. Correspondingly, a quadratic regression approximation model for the reduced Nusselt number $Nn_{x}$ has also been described for 100 varying values of thermal slip parameter *D* and radiation parameter $Tr$, obtained indiscriminately from the intervals $\left[ {0.1\,,\,\,0.7} \right]$ and $\left[ {0.05,\,\,0.80} \right]$ separately for the rising values of Eckert number *Ec*. The other enduring parameters are considered constant as reported in the previous section during the approximation process.

The quadratic regression approximation model for the estimated $Sf_{x}$ owing to change in suction parameter *S* and velocity slip parameter *L* is given by

20 $$Sf_{x\,(est)} = Sf_{x} + b_{1} S + b_{2} L + b_{3} S^{2} + b_{4} L^{2} + b_{5} SL,$$

whereas, quadratic regression approximation formula for the estimated $Nn_{x}$ due to variation in thermal slip parameter *D* and radiation parameter $Tr$ is represented as

21 $$Nn_{{x\,\left( {est} \right)}} = Nn_{x} + c_{1} D + c_{2} (Tr) + c_{3} D^{2} + c_{4} (Tr)^{2} + c_{5} D(Tr),$$

where $b_{1} ,\,\,b_{2} ,\,\,b_{3} ,\,\,b_{4} ,\,\,b_{5}$ and $c_{1} ,\,\,c_{2} ,\,\,c_{3} ,\,\,c_{4} ,\,\,c_{5}$ are respectively the coefficients of the quadratic regression approximation model for the reduced $Sf_{x}$ and $Nn_{x}$.

Tables 6 and 7 demonstrate the coefficients of quadratic regression approximated values of $Sf_{x}$ and $Nn_{x}$ respectively for different enduring parameters. The optimum relative error bounds $\varepsilon \,{\text{and}}\,\varepsilon_{1}$ for $Sf_{x}$ and $Nn_{x}$ have also been analysed by using the relations $\varepsilon = \left| {Sf_{x\,(est)} - Sf_{x} } \right|/Sf_{x}$ and $\varepsilon_{1} = \left| {Nn_{x\,(est)} - Nn_{x} } \right|/Nn_{x}$ respectively. These error bounds are mentioned in Tables 6 and 7. It is worthy to remark that as the strength of the magnetic field or viscous dissipation effect improves, the coefficients of *S* or *Tr* becomes negative as observed from Tables 6 and 7, respectively. This finding reveals that the suction and thermal radiation have an adverse influence on the approximated skin friction coefficients and wall temperature gradients respectively. Moreover, from the tabulated values it is found that the coefficients of the velocity slip parameter are greater than those of the suction parameter in magnitude; which reveals the findings that a small variation in the velocity slip parameter *L* results in a significant change in the shear stress function in comparison to the suction parameter *S*. Likewise, a small augmentation in the thermal slip factor causes a considerable variation in the heat transport rate in comparison to the thermal effect. In addition, it is observed that the optimum relative error of quadratic regression approximation for the reduced wall temperature gradient is approximately zero, and the approaching rate towards this admirable accuracy level is faster than that of the quadratic regression approximation for the reduced skin friction coefficients.

**Table 6:** Quadratic regression approximated coefficients of $Sf_{x}$ owing to variations in *S* and *L* and optimum relative error bound $\varepsilon$ are tabulated as mentioned below.

*M*	$Sf_{x}$	${b}_{1}$	${b}_{2}$	${b}_{3}$	${b}_{4}$	${b}_{5}$	$\varepsilon$
0.5	− 0.9763	− 0.4911	1.4216	0.0136	− 1.1786	0.5344	0.0125
1.5	− 1.1097	− 0.4060	1.6048	0.0308	− 1.2562	0.4880	0.0144
2.5	− 1.2006	− 0.4460	1.8042	0.0208	− 1.4661	0.5127	0.0077
3.5	− 1.2938	− 0.3564	1.8335	0.0368	− 1.3291	0.4544	0.0060

**Table 7:** Quadratic regression approximated coefficients of $Nn_{x}$ owing to variations in $D$ and *Tr* and also optimum relative error bound $\varepsilon_{1}$ are obtained as under.

$Ec$	$Nn_{x}$	${c}_{1}$	${c}_{2}$	${c}_{3}$	${c}_{4}$	${c}_{5}$	*ε*_*1*_
0.04	− 1.4635	2.0210	− 0.4476	− 1.6153	0.0881	− 0.1500	0.0021
0.10	− 1.3098	1.8087	− 0.4229	− 1.4882	0.0728	− 0.0924	0.0024
0.16	− 1.1588	1.6332	− 0.3970	− 1.4195	0.0626	− 0.0831	0.0030
0.22	− 1.0032	1.4118	− 0.3805	− 1.2201	0.0570	− 0.0345	0.0035

## Conclusions

In this research study, due to the remarkable significance in the advancement of robust apparatus used in the energy sector, nuclear power station, medical sciences, satellites, sensing outlets, gas turbines and supercapacitors, etc., numerical and statistical explorations have been accomplished to capture the flow features of the dynamics of ethylene glycol-based hybrid nanofluid containing graphene and MoS_2_ nanoparticles over an exponentially stretchable sheet with partial slip and thermal jump conditions. Some important conclusions of the study are highlighted below:

- The fluid motion of hybrid nanofluid is strongly retarded by increasing the strength of the magnetic field and angle of the aligned magnetic field however leave a reversal impact on the temperature of the hybrid nanofluid
- Both the temperature and velocity dispersal profiles are enhanced for improving the injection parameter while the suction parameter has reversed impact on the profiles
- The temperature of hybrid nanofluid can be augmented by improving viscous dissipation and thermal effects while it can be reduced owing to a rise in the values of heat absorption parameter and thermal slip factor
- The shear stress functional values can be enhanced due to the improvement in the suction, angle of aligned magnetic field and magnetic field effects while it can be reduced owing to rise in injection parameter at the surface of the stretchable sheet under both the situation of velocity slip and no-slip
- The heat transport rate can be improved by the inspirations of heat absorption, suction and thermal radiation while the angle of an aligned magnetic field, viscous dissipation, magnetic field, injection and thermal slip factor are significant to reduce the heat transport rate of hybrid nanofluid
- The quadratic regression approximation analysis discloses that the suction and thermal radiation have an adverse influence on the approximated skin friction coefficients and wall temperature gradients. Further, a small variation in the velocity slip parameter results in a significant change in the shear stress function in comparison to the suction parameter. Likewise, a small augmentation in the thermal slip factor causes a considerable variation in the heat transport rate in comparison to the thermal effect.
- The optimum relative error of quadratic regression approximation for the reduced wall temperature gradient is approximately zero, and the approaching rate towards this admirable accuracy level is faster than that of the quadratic regression approximation for the reduced skin friction coefficients.
